# Autoimmune myelofibrosis secondary to systemic lupus erythematosus: a diagnostic challenge in a resource-limited setting

**DOI:** 10.1093/omcr/omag139

**Published:** 2026-07-27

**Authors:** Abbas Godian, Noorein Omar, Zainab Malingumu, Basil Tumaini, Elizabeth Msangi

**Affiliations:** Department of Internal Medicine, Muhimbili University of Health and Allied Sciences, 9 United Nations Road, Upanga West, Ilala District, P. O. Box 65001, 11103, Dar es Salaam, Tanzania; Department of Internal Medicine, Muhimbili University of Health and Allied Sciences, 9 United Nations Road, Upanga West, Ilala District, P. O. Box 65001, 11103, Dar es Salaam, Tanzania; Department of Internal Medicine, Muhimbili University of Health and Allied Sciences, 9 United Nations Road, Upanga West, Ilala District, P. O. Box 65001, 11103, Dar es Salaam, Tanzania; Department of Internal Medicine, Muhimbili University of Health and Allied Sciences, 9 United Nations Road, Upanga West, Ilala District, P. O. Box 65001, 11103, Dar es Salaam, Tanzania; Department of Internal Medicine, Muhimbili University of Health and Allied Sciences, 9 United Nations Road, Upanga West, Ilala District, P. O. Box 65001, 11103, Dar es Salaam, Tanzania

**Keywords:** autoimmune myelofibrosis, systemic lupus erythematosus, pancytopenia, bone marrow failure, sub-Saharan Africa, diagnostic challenge

## Abstract

Autoimmune myelofibrosis (AIMF) is a rare, potentially reversible cause of bone marrow failure often linked to systemic autoimmune disease. We report a diagnostically challenging case of a 26-year-old woman from sub-Saharan Africa who presented with transfusion-dependent pancytopenia and an initial dry-tap marrow, with further testing limited by financial constraints. Seven months later, evolving clinical and immunologic features—including fever, leukopenia, thrombocytopenia, malar rash, positive ANA, and a sterile exudative pleural effusion—fulfilled the 2019 EULAR/ACR criteria for systemic lupus erythematosus (SLE). AIMF secondary to SLE was subsequently diagnosed, and she demonstrated a rapid hematologic response to glucocorticoids and hydroxychloroquine, remaining transfusion-independent for over five months. This case underscores the diagnostic complexity of AIMF in resource-limited settings and highlights the importance of sustained clinical suspicion and adaptable diagnostic strategies when evaluating unexplained pancytopenia.

## Introduction

Autoimmune myelofibrosis (AIMF) is an uncommon, steroid-responsive, non-clonal marrow fibrosis most often associated with systemic autoimmune disease, particularly systemic lupus erythematosus (SLE) [1]. In contrast to primary myelofibrosis (PMF)—a clonal myeloproliferative neoplasm characterized by atypical megakaryocytes, leukoerythroblastosis, and JAK2/CALR/MPL mutations, and typically lacking responsiveness to immunosuppressive therapy [2]—AIMF generally demonstrates prompt haematologic improvement with immunosuppression [2, 3]. Although up to 70% of AIMF cases occur in the context of SLE, the condition remains under-recognized, especially in young women presenting with pancytopenia before overt autoimmune features emerge [3]. Diagnostic uncertainty arises because AIMF clinically resembles aplastic anaemia, myelodysplastic/myeloproliferative neoplasms, and nutritional deficiencies, and in resource-limited settings this challenge is compounded by limited access to confirmatory tests.

## Case report

A 26-year-old woman with no prior chronic illness presented with a one-year history of transfusion-dependent anaemia characterized by fatigue, palpitations, exertional intolerance, and intermittent low-grade fevers. She had received over 13 units of packed red blood cells without any history of bleeding, autoimmune disease, or relevant family history. Examination revealed pallor without rashes, arthritis, or lymphadenopathy.

**Figure 1:**
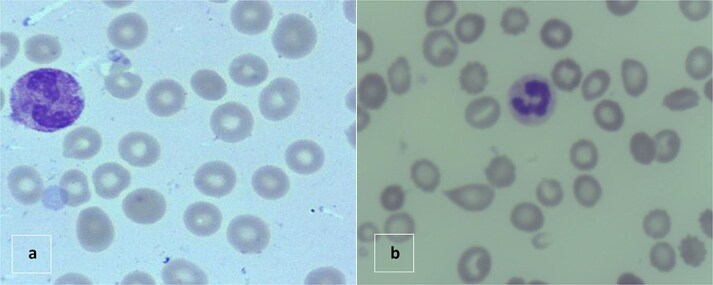
Serial peripheral blood smears demonstrating persistent pancytopenia. (a) Initial smear on first admission showing pancytopenia with marked anisocytosis, target cells, and a neutrophil with toxic granulation. (b) Follow-up smear on 3 January 2025 showing severe pancytopenia with pronounced anisopoikilocytosis, tear-drop cells, schistocytes, hypochromic cells, occasional macrocytes, target cells, echinocytes, and acanthocytes.

**Figure 2:**
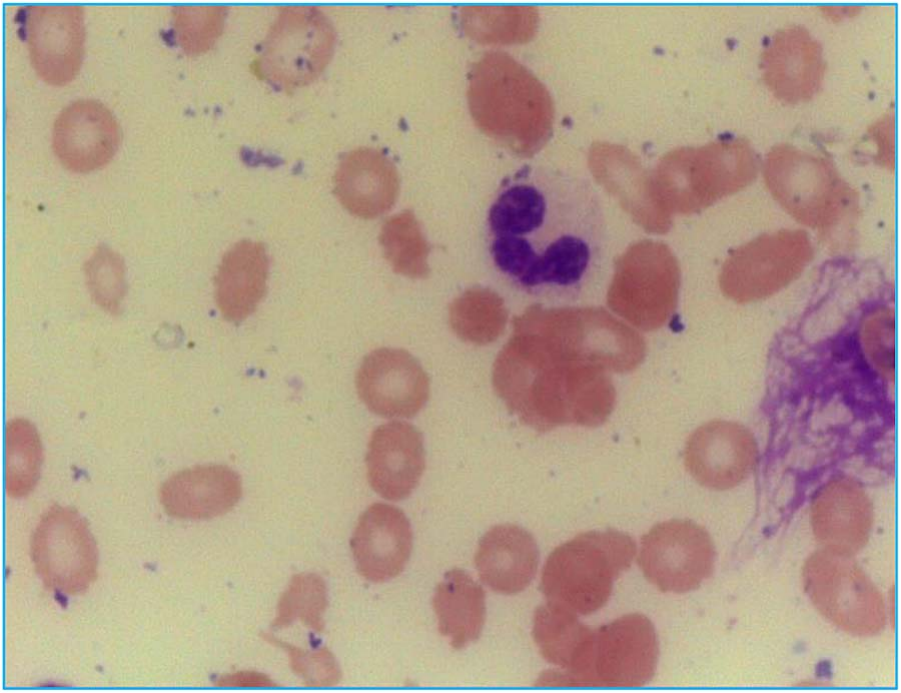
Bone marrow aspirate demonstrating hypocellularity. Low-yield, aparticulate aspirate smear in a young woman with chronic transfusion-dependent anaemia. No definitive diagnostic features were observed, prompting further investigation with a trephine biopsy.

Initial investigations showed pancytopenia with a persistently low reticulocyte count (Table 1, Fig. 1). A provisional diagnosis of aplastic anaemia was considered; however, bone marrow evaluation was deferred due to financial constraints. Three months later, she re-presented with severe anaemia. Bone marrow aspiration yielded a dry tap (Fig. 2), and trephine biopsy demonstrated stromal spindle-cell proliferation consistent with marrow fibrosis. Empirical nutritional supplementation, including intramuscular vitamin B12, did not produce improvement.

**Table 1:** Serial haematological and biochemical parameters during multiple admissions in a young woman with pancytopenia.

Parameter	Date/Result						Reference range
	03/04/2024	25/05/2024	11/08/2024	26/12/2024	03/01/2025	24/04/2025	
Leucocytes (× 10^9^/l)	1.02	0.58	0.43	2.13	0.67	1.92	4–11
Neutrophils	0.52	0.11	0.15	1.59	0.56	1.16	2.0–7.0
Lymphocytes	0.47	0.29	0.26	0.24	0.08	0.45	1.5–4.0
Monocytes	0.03	0.17	0.02	0.22	0.03	0.25	0.2–0.8
Eosinophils	0.002	0.000	0.000	0.001	0.000	0.01	0.04–0.4
Basophils	0.001	0.010	0.000	0.080	0.000	0.05	0.01–0.1
Erythrocytes (× 10^12^/l)	1.16	1.54	2.4	2.1	1.32	4.41	3.5–5.5
Hb (g/dl)	3.42	3.33	6.0	5.4	3.2	10.3	11–16
HCT (%)	11.14	12.05	20.6	17.2	11.3	36.3	33–54
MCV (fL)	80.2	78.25	85.2	82.1	85.3	82.3	80–100
MCH (pg)	22.4	21.6	25.1	26.0	24.4	23.3	27–34
MCHC (g/dl)	28.3	27.6	29.5	31.7	28.6	26.3	32–36
RDW (%)	18.1	24.8	20.4	17.6	15.3	18.3	11–16
Platelet count (× 10^9^/l)	11.4	17.3	21.1	20.1	19.8	65.9	100–450
Reticulocyte count (× 10^9^/l)	10.1	11.9	18.5	5.4	10.0		25–100
Ferritin (μg/l)		225		3068	2000		10–250
Serum iron (μmol/l)				19.8			10–30
Transferrin saturation (%)				97	47		20–50
Serum folate level (nmol/l)		10.4		9.9	12.3		7–45
Serum vitamin B_12_ level (pmol/l)		162		287	509		200–800
Lactate dehydrogenase (U/l)	247		170				125–220
Indirect bilirubin (μmol/l)					11.1		3.4–12.0
Haptoglobin (g/l)					0.9		0.4–2.4

The persistently low reticulocyte count supported a hypoproliferative process.

Hb: haemoglobin level; HCT: haematocrit; MCH: mean corpuscular haemoglobin; MCHC: mean corpuscular haemoglobin concentration; MCV: mean corpuscular volume; RDW: red cell distribution width.

Seven months after initial presentation, she developed progressive abdominal distension, dyspnoea, facial erythema, and weight loss. Examination revealed malar rash, marked pallor, angular stomatitis, splenomegaly, ascites, and bilateral pleural effusion which was later confirmed to be sterile and exudative. Serial complete blood counts confirmed profound pancytopenia (haemoglobin nadir 3.2 g/dL; leucocytes 0.43 × 10^9^/L; platelets 11.4 × 10^9^/L) with reticulocytopenia. Peripheral smears demonstrated anisopoikilocytosis, tear-drop cells, target cells, schistocytes, echinocytes, and acanthocytes, without blasts or dysplasia.

**Figure 3:**
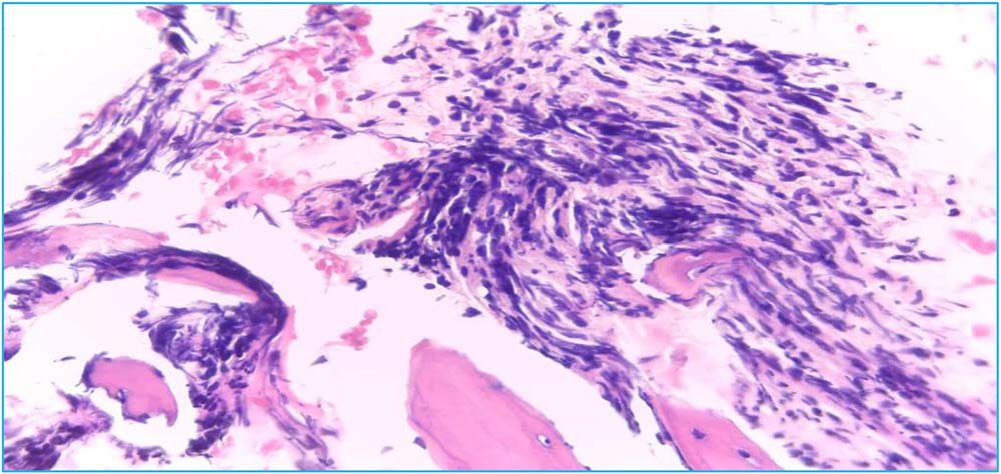
Trephine biopsy demonstrating marrow fibrosis. Hematoxylin and eosin–stained section shows a markedly hypocellular, aparticulate marrow with proliferation of spindle-shaped stromal cells, consistent with fibrotic transformation. The myeloid and erythroid series are markedly reduced, without evidence of dysplasia. Megakaryocytes are sparse and non-atypical. No osteosclerosis, lymphoid aggregates, granulomas, or infiltrative lesions are seen. Reticulin staining and molecular studies could not be performed due to resource limitations.

Trephine biopsy showed a markedly hypocellular, aparticulate specimen with stromal spindle-cell proliferation (Fig. 3). Reticulin staining and molecular studies (JAK2/CALR/MPL) were unavailable. Autoimmune testing revealed positive antinuclear antibody and direct antiglobulin test, with anti-centromere protein B and anti-Ku antibodies detected (Table 2). Viral serologies (HBsAg, anti-HCV, HIV ELISA) were negative; parvovirus B19 testing was unavailable. Haemolysis workup showed mildly elevated LDH with normal indirect bilirubin and haptoglobin. Thyroid function, renal function, electrolytes, and urinalysis were normal. Tuberculosis evaluation—including sputum culture and ADA level—was negative. Imaging confirmed splenomegaly (21.2 cm), pleural effusion, and ascites; liver ultrasound was normal.

**Table 2:** Autoantibody profile results from initial immunological workup.

Autoantibody test	Result status
Antinuclear antibody (ANA)	Positive
Anti-double stranded DNA antibody (dsDNA)	Equivocal
Anti-nucleosome antibody	Negative
Anti-histone antibody	Negative
Anti-Smith D1 antibody (SmD1)	Negative
Anti-proliferating cell nuclear antigen antibody (PCNA)	Negative
Anti-ribosomal P protein antibody (RPP)	Negative
Anti-Sjögren’s-syndrome-related antigen A (60 kDa) {Ro60 (SSA-60)}	Negative
Anti-Sjögren’s-syndrome-related antigen A (52 kDa) {Ro52 (SSA-52)}	Negative
Anti-Sjögren’s-syndrome-related antigen B {La (SSB)}	Equivocal
Anti-centromere protein B antibody (CENP-B)	Positive
Anti-topoisomerase I antibody (Scl-70)	Negative
Anti-U1 small nuclear ribonucleoprotein antibody (U1-snRNP)	Negative
Anti-mitochondrial antibody M2 (AMA-M2)	Negative
Anti-histidyl-tRNA synthetase antibody (Jo-1)	Negative
Anti-polymyositis/scleroderma antibody (PM-Scl)	Negative
Anti-Mi-2 antibody	Negative
Anti-Ku antigen antibody	Positive

Alternative causes of marrow failure were systematically excluded. Aplastic anaemia was unlikely due to stromal fibrosis and the emergence of autoimmune features. Primary myelofibrosis and other myelodysplastic/myeloproliferative neoplasms were unsupported by the absence of atypical megakaryocytes or leukoerythroblastosis. Nutritional deficiencies were ruled out by normal folate and vitamin B12 levels and lack of response to supplementation. Thrombotic microangiopathy and hemophagocytic lymphohistiocytosis were excluded based on absent haemolysis, renal dysfunction, classic organomegaly, or very high ferritin levels.

Diagnostic clarity emerged when evolving clinical features—malar rash, fever, leukopenia, thrombocytopenia—and a sterile exudative pleural effusion, together with positive ANA, yielded a cumulative score of 18 on the 2019 EULAR/ACR SLE classification criteria, although complement levels were unfortunately unavailable. AIMF secondary to SLE was therefore diagnosed.

Due to clinical deterioration and resource limitations, empiric immunosuppression was commenced. She received intravenous methylprednisolone 1 g daily for three days, followed by oral prednisolone 50 mg/day tapered over six weeks to 5 mg/day, in addition to hydroxychloroquine 200 mg twice daily. Within three weeks, she showed marked clinical and haematologic recovery, including reduction of spleen size to 16 cm and stabilization of haemoglobin (Table 1). She has remained transfusion-independent for over five months and continues follow-up in rheumatology and haematology clinics. At her next rheumatology–haematology review in approximately one month, mycophenolate mofetil will be initiated as a steroid-sparing immunosuppressant while continuing hydroxychloroquine and gradually tapering prednisolone.

## Discussion

Autoimmune myelofibrosis (AIMF) is an uncommon, potentially reversible, non-clonal marrow fibrosis linked to SLE. Although bone marrow abnormalities in SLE are well documented, overt myelofibrosis occurs in fewer than 1% of cases [4]. AIMF accounts for 10–15% of myelofibrosis presentations yet remains under-recognized in resource-limited settings, where lack of reticulin staining, immunohistochemistry, and molecular testing complicates differentiation from primary myelofibrosis (PMF).

The patient’s initial presentation—profound pancytopenia, a dry-tap aspirate, and absence of autoimmune features—raised suspicion for multiple marrow-failure syndromes. Trephine biopsy demonstrated stromal spindle-cell proliferation supportive of fibrosis, but inability to perform reticulin or JAK2/CALR/MPL mutation analysis limited early classification. These constraints mirror real-world barriers in sub-Saharan Africa.

Clinical evolution later provided diagnostic clarity. The delayed onset of lupus features—malar rash, fever, leucopenia, thrombocytopenia, and a sterile exudative pleural effusion—fulfilled the 2019 EULAR/ACR SLE classification criteria. Similar to published AIMF series, our patient was a young woman with severe pancytopenia and brisk corticosteroid responsiveness, consistent with observations from the French multicentre cohort and Mayo Clinic series [3, 5]. However, the degree of splenomegaly was unusual. Potential mechanisms include extramedullary haematopoiesis from fibrotic marrow, SLE-related immune hyperplasia, and transfusion-related hypersplenism. Its marked reduction after steroids strongly supports an autoimmune rather than clonal myeloproliferative process.

Distinguishing AIMF from PMF is critical. PMF typically exhibits atypical megakaryocytes, leukoerythroblastosis, and clonal driver mutations [2], none of which were present here. The absence of dysplastic megakaryocytes and leukoerythroblastosis, together with steroid-induced hematologic recovery, favoured AIMF secondary to SLE.

Glucocorticoid responsiveness remains the hallmark of AIMF [3]. Our patient’s rapid clinical and hematologic improvement with high-dose methylprednisolone and tapering prednisolone mirrors reported outcomes, with most patients achieving partial or complete remission. Nonetheless, long-term data remain limited, especially in African contexts where follow-up is variable. Continued surveillance is essential to detect relapse and mitigate glucocorticoid toxicity [5].

Overall, this case underscores the importance of maintaining suspicion for AIMF in unexplained pancytopenia, employing stepwise exclusion of alternative diagnoses, and recognizing that evolving autoimmune features may confirm the diagnosis despite initial limitations in diagnostic testing.
